# Intracellular Transport and Cytotoxicity of the Protein Toxin Ricin

**DOI:** 10.3390/toxins11060350

**Published:** 2019-06-18

**Authors:** Natalia Sowa-Rogozińska, Hanna Sominka, Jowita Nowakowska-Gołacka, Kirsten Sandvig, Monika Słomińska-Wojewódzka

**Affiliations:** 1 Department of Medical Biology and Genetics, Faculty of Biology, University of Gdańsk, Wita Stwosza 59, 80-308 Gdańsk, Poland; natalia.sowa@phdstud.ug.edu.pl (N.S.-R.); hanna.sominka@phdstud.ug.edu.pl (H.S.); jowita.nowakowska@phdstud.ug.edu.pl (J.N.-G.); 2 Department of Molecular Cell Biology, Institute for Cancer Research, Oslo University Hospital, 0379 Oslo, Norway; kirsten.sandvig@ibv.uio.no; 3 Faculty of Mathematics and Natural Sciences, Department of Biosciences, University of Oslo, 0316 Oslo, Norway

**Keywords:** ricin, protein synthesis inhibition, apoptosis

## Abstract

Ricin can be isolated from the seeds of the castor bean plant (*Ricinus communis*). It belongs to the ribosome-inactivating protein (RIP) family of toxins classified as a bio-threat agent due to its high toxicity, stability and availability. Ricin is a typical A-B toxin consisting of a single enzymatic A subunit (RTA) and a binding B subunit (RTB) joined by a single disulfide bond. RTA possesses an RNA N-glycosidase activity; it cleaves ribosomal RNA leading to the inhibition of protein synthesis. However, the mechanism of ricin-mediated cell death is quite complex, as a growing number of studies demonstrate that the inhibition of protein synthesis is not always correlated with long term ricin toxicity. To exert its cytotoxic effect, ricin A-chain has to be transported to the cytosol of the host cell. This translocation is preceded by endocytic uptake of the toxin and retrograde traffic through the trans-Golgi network (TGN) and the endoplasmic reticulum (ER). In this article, we describe intracellular trafficking of ricin with particular emphasis on host cell factors that facilitate this transport and contribute to ricin cytotoxicity in mammalian and yeast cells. The current understanding of the mechanisms of ricin-mediated cell death is discussed as well. We also comment on recent reports presenting medical applications for ricin and progress associated with the development of vaccines against this toxin.

## 1. Introduction

The toxin ricin is a naturally occurring, extremely toxic protein isolated from the seeds of the castor plant, *Ricinus communis*. This toxin was first described in 1888 by Peter Hermann Stillmark, who identified the active ingredient isolated from the castor seeds as a protein [1]. Interestingly, he described in his doctoral thesis, ricin’s agglutinating properties, being the first person who defined and characterized a specific carbohydrate-binding protein, that later was called lectin [2]. At that time, the ability of extracts from *Ricinus communis* to agglutinate erythrocytes and to precipitate serum proteins was considered to be the mechanism behind the cytotoxic action of ricin. Later experiments showed that the toxicity and agglutination effects are separate properties of this toxin [3]. The structure of ricin was described by Olsnes and Pihl [4]. They also found that ricin exerts its cytotoxic effect by enzymatic action on eukaryotic ribosomes resulting in inhibition of protein synthesis [3,4,5]. Considering the mechanism of ricin cytotoxic activity, it was the very first identified RIP (ribosome-inactivating protein) assigned to the class II of this group of protein toxins [6,7,8].

**Figure 1:**
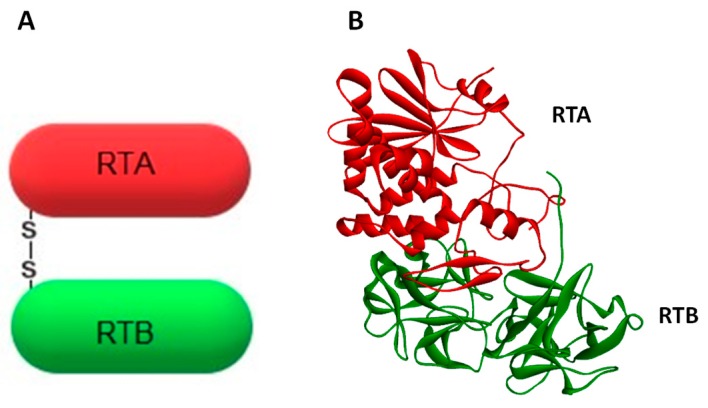
Schematic representation (**A**) and crystal structure (**B**) of the toxin ricin. The enzymatically-active subunit (A-chain) is marked in red, whereas the binding domain (B-chain) is presented in green. Both subunits are linked by a single disulfide bond. Crystal structure has been obtained from the PDB protein data bank (code 2AA1).

Ricin belongs to the A-B family of protein toxins. The A-chain (RTA, ~32 kDa) is linked by a single disulfide bond to the lectin B-chain (RTB, ~34 kDa). Together they form ricin holotoxin (Figure 1). RTA possesses an enzymatic activity [6,7], whereas RTB binds to eukaryotic cell-surface glycoproteins and glycolipids [9]. Ricin A-chain specifically depurinates the α-sarcin-ricin loop (SRL) by hydrolyzing the N-glycosidic bond at adenine 4324 located at a GAGA hairpin of SRL of the 28S rRNA in the eukaryotic large ribosomal subunit [5], (for review see Refs. [8,10,11]). Interestingly, ricin does not remove an adenine from rRNA in whole *E. coli* ribosomes, thus genes coding for ricin could be expressed in *E. coli* [12]. It is considered that the sarcin-ricin loop is one the largest universally-conserved regions of the ribosome [13,14]. This highlights its importance in ribosome function. Indeed, SRL significantly influences the proper assembly of the functional structure of the 50S prokaryotic subunit [15], and it is highly probable that this loop fulfills a similar role in the large ribosomal subunit in eukaryotic cells. However, what is most important for ricin toxicity is that depurination of SRL prevents the binding of two crucial factors operating in the machinery of protein synthesis: the eukaryotic elongation factor 1 (eEF-1) and the elongation factor 2 (eEF-2) [9,16,17]. This blocks protein synthesis and is a prerequisite for the cytotoxic effect of ricin. A single ricin A-chain molecule is able to inactivate approximately 1500 ribosomes per minute [18,19]. It happens much faster than the cell can produce new ones [20]. Ricin’s lethal dose in humans was estimated to be about 1.78 mg for an average adult [21]. However, its toxicity depends on the route of exposure. Inhalation is more potent than oral administration. The inhalation median lethal dose (LD50) is 3–5 µg/kg, while the oral LD50 is 20 mg/kg [22]. Due to ricin’s high toxicity and stability, ease of production and good availability, it has been classified by the US Centers for Disease Control and Prevention (CDC) as a Category B Select Agent. Implementation of the Chemical Weapons Convention (CWC) in the national legislation of the 192 signatory countries (June 2017) makes undeclared ricin purification a global crime [23]. Despite the fact that ricin-mediated depurination of rRNA has been quite well described, other mechanisms involved in its cytotoxicity are not completely clarified. In fact, the inhibition of protein synthesis by ricin A-chain is not exclusively responsible for the cytotoxic effect of this toxin [24]. It has been demonstrated that ricin can induce apoptosis, cell membrane damage, membrane structure and function alteration, and release of cytokine inflammatory mediators [25,26,27,28,29,30]. In general, the inhibition of protein synthesis seems to precede apoptosis and be necessary for this event. It was, however, suggested that two different motifs present in ricin A-chain may be involved in ricin-mediated inhibition of protein synthesis and apoptosis [31,32] and that B-chain in human myeloid leukemia cells (U937) is able to induce apoptosis through its lectin activity without the contribution of the A-chain [33]. 

Elucidation of the entire mechanisms of ricin toxicity is crucial to fully utilize, but also to control all properties of this toxin. Ricin is being considered as one of the most toxic substances that exists. It can be used as a potential tool in bioterrorist attacks [34,35]. Thus, the development of effectively working antitoxin agents is of particular interest [36,37,38,39]. On the other hand, ricin conjugated with specific antibodies, other proteins, peptides or nanoparticles can be selectively directed to target cells. This ensures the possibility of a huge application of this toxin in medicine [40,41,42,43,44]. In this review, we describe the most important steps of ricin intracellular transport as well as diverse and complicated mechanisms of its action on cells. We also summarize the newest reports concerning the development of vaccines against ricin and biomedical applications of this toxin.

## 2. Intracellular Transport of Ricin

### 2.1. Uptake of Ricin into the Cell

Ricin B-chain recognizes complex types of carbohydrate receptors present on the surface of eukaryotic cells. These receptors contain either terminal N-acetylgalactosamine or β-1,4-linked galactose residues [9]. Galactosyl-residues are abundant on the surface of most cell types, thus the majority of eukaryotic cells are sensitive to ricin. Moreover, this toxin can also bind the mannose-type glycans on cells that carry those receptors i.e., macrophages or rat liver endothelial cells [45]. This is due to the fact that both the A- and B-chains of ricin are glycoproteins that contain mannose-rich oligosaccharides. However, in contrast to galactosyl-residues, most cell types do not express mannose receptors and consequently ricin in such cells can be internalized exclusively by galactosyl moieties. It is assumed that 10^6^–10^8^ ricin molecules can be bound to the cell surface [46]. Human cervical cancer cells (HeLa) for example contain 3 × 10^7^ ricin-binding sites per cell [47], although not all of these sites are involved in toxin uptake.

**Figure 2:**
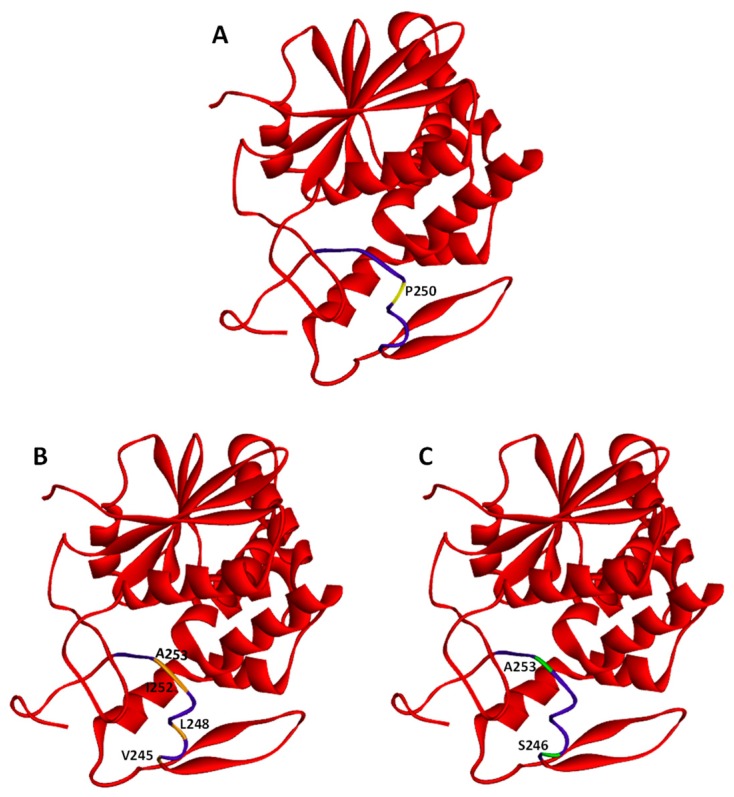
Crystal structures of ricin A-chain. PDB protein data bank (code 2AA1). Whole ricin A-chain (RTA) structures are marked in red. The hydrophobic region (Val245 to Val256) is indicated in purple. Several residues have been selected within the hydrophobic region and modified to obtain RTA with changed secondary structure or changed hydrophobicity. These residues are: (**A**) P250 (marked in yellow) has been modified (P250A) to produce RTA with a changed secondary structure; (**B**) V245, L248, I252, and A253 (marked in orange) have been modified (V245S, L248N, I252N, A253S) to obtain RTA with decreased hydrophobicity (RTA DHF); (**C**) S246 and A253 (marked in green) have been changed (S246V, A253V) to obtain RTA with increased hydrophobicity (RTA IHF).

**Figure 3:**
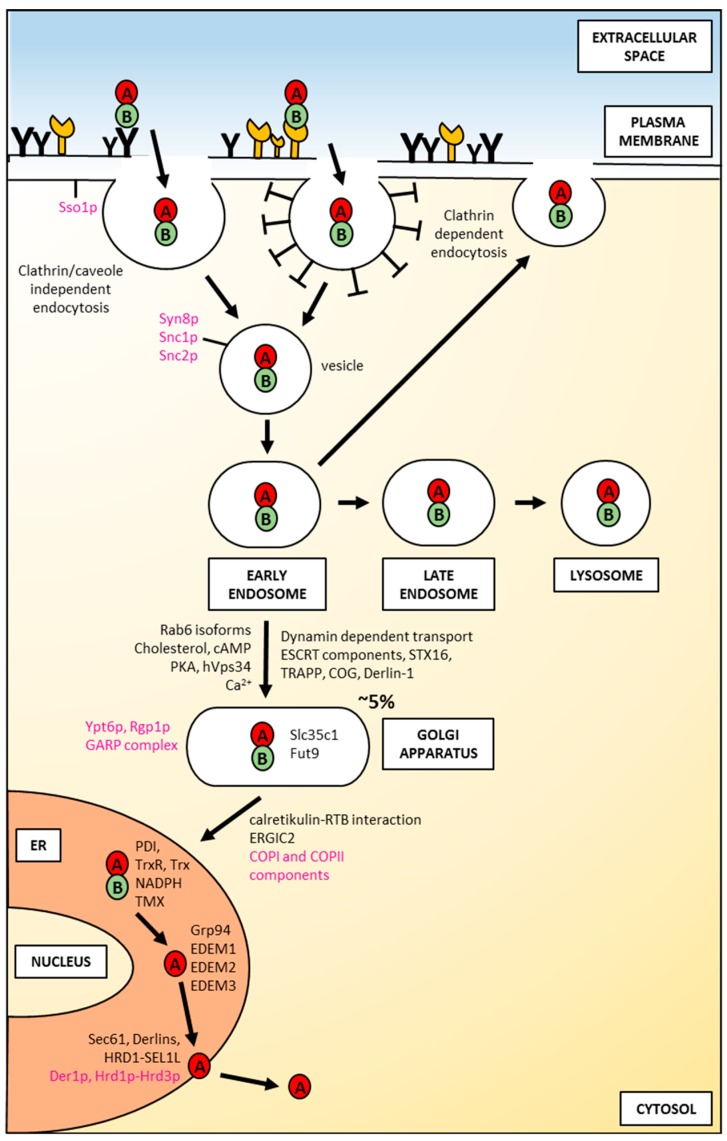
An overview of factors involved in the intracellular transport of ricin in mammalian and yeast cells. Yeast proteins are shown in magenta; mammalian proteins are shown in black. For a detailed description, please see the text.

After binding of ricin to the cell-surface receptors, the holotoxin is transported into the cell by endocytosis. It has been demonstrated that ricin is able to employ different endocytic mechanisms, which are believed to be mainly connected with the fact that it can recognize and bind to a great variety of cell surface components. It is even suggested that ricin can utilize all types of endocytic mechanisms that operate in a given cell [48]. First studies of ricin uptake demonstrated that it can be internalized by clathrin-dependent endocytosis in Vero cells (African green monkey kidney cells) [49]. However, further experiments showed that the inhibition of clathrin-coated pits formation in Hep-2 cells (derivative of HeLa) did not change ricin cytotoxicity [50] and that generally it can be taken into cells from non-coated parts of the cell surface membrane [51]. Additional evidence that ricin can be endocytosed by clathrin-independent mechanisms comes from experiments in which a dominant negative mutant of dynamin, a protein that is required for clathrin-related endocytosis, was used. The overexpression of the dynamin defective in GTP binding and hydrolysis in COS-7y cells (derived from kidney tissue of the African green monkey) did not alter cell sensitivity to ricin [52,53]. However, it should be noted that the expression of the same mutant dynamin (K44A) in HeLa cells inhibited ricin toxicity (see below). Moreover, it has been demonstrated that cholesterol plays an important role in endocytosis by clathrin-coated pits [54]. However, endocytosis of ricin was not changed significantly after cholesterol extraction from the membrane [54]. Effective extraction of cholesterol also disrupts other structures called caveolae that can be involved in clathrin-independent endocytosis [54,55], but independent experiments showed that caveolae were not necessary for ricin endocytosis [52]. Importantly, cholesterol is required for other endocytic mechanisms as well [56]. Thus, endocytic uptake of ricin can be both clathrin- and caveolae-independent [57]. Binding and endocytosis of ricin can be related to specific features of this toxin. Mutation that changes the secondary structure of its A-chain into a more helical structure (Figure 2A) has no influence on binding or endocytosis of modified holotoxin [58]. However, mutations that modify hydrophobicity of RTA (Figure 2B,C) significantly affect the binding of the altered holotoxins to the cell membrane [59]. A GFP-based reporter assay has very recently been applied in order to identify cellular components required for RTA intracellular transport in yeast [60]. The data indicated that proteins Syn8p, Sso1p and Snc1p influence ricin A-chain trafficking [60] (Figure 3). Syn8p and Snc1p form a complex that plays a role in protein uptake from the plasma membrane to endosomes [61]. Sso1p is a plasma membrane t-SNARE protein that together with v-SNARE Snc2p are required for the fusion of Golgi-derived vesicles with the plasma membrane [62]. It is unknown whether mammalian homologues of these proteins are also involved in ricin endocytosis.

### 2.2. Intracellular Routing of Ricin to the Endoplasmic Reticulum

After endocytosis ricin traffics to early endosomes (Figure 3). It has been demonstrated, that at this stage, the majority of toxin can be transported back to the cell surface in an apparently intact form [63]. In early endosomes, ricin also starts to be degraded [64]. This proteolysis is continued after its predominant transport to late endosomes and lysosomes [65] (Figure 3). Interestingly, efficient transport to lysosomes depends on how ricin is internalized. Significantly more toxin was transferred from endosomes to lysosomes upon internalization by mannose receptors in comparison to uptake by galactosyl-residues [45]. It was suggested that the different stabilities of ricin at endosomal low pH may be characteristic for these two binding mechanisms. This would explain the observed differences in the transport from endosomes to lysosomes between the two internalization pathways. In addition, as already mentioned in this review, modified ricin with a changed secondary structure that carries a point mutation in the hydrophobic region of the C-terminal A-chain (P250A) (Figure 2A) is more extensively degraded in endosomes/lysosomes than the wild-type toxin [57]. Both cathepsin B and cathepsin D were found to be involved in increased P250A ricin degradation. It cannot be excluded that P250 holotoxin has a somehow altered conformation in comparison to the wild-type toxin. This altered conformation might influence its endosomal/lysosomal degradation. It was visualized by electron microscopy that only about 5% of endocytosed ricin is transported to the tran*s*-Golgi network (TGN) [66,67]. This transport proceeds directly from early endosomes, excluding the late endosomes-tran*s*-Golgi pathway (Figure 3) [68]. From the Golgi complex, ricin is moved to the endoplasmic reticulum (ER). Ricin transport to the Golgi network and to the ER were confirmed experimentally [66,67,69]. Both pathways, endosome to Golgi and retrograde movement from the Golgi to the ER can engage complicated mechanisms of ricin intracellular transport. Brefeldin A is an important drug in these studies, because it disturbs the Golgi complex [70]. In cells treated with this compound, ricin transport was completely inhibited, and cell intoxication was blocked [71,72]. Interestingly, this was observed only in cells where the Golgi complex was sensitive to brefeldin A (BFA) [72].

In cells resistant to BFA, the polarized kidney epithelial cell line MDCK, BFA sensitized cells to ricin [73]. This drug does not change Golgi morphology in these cells and thus, it would be possible that increased transport to the Golgi apparatus is responsible for higher toxicity of ricin in MDCK cells. However, this did not seem to be the case. Moreover, it was demonstrated that BFA affects endosomal structures in those cells [73].

Ricin transport to the Golgi was also demonstrated by adding a short amino acid stretch, a tyrosine sulfation site that comes from rat cholecystokinin precursor. This extra stretch was added to the C-terminal end of the RTA, creating the ricin-A-sulf-1 [69]. When such a modified toxin was delivered to the Golgi complex, sulfotransferases that are specific enzymes for this compartment [74], catalyse the addition of sulfate to ricin. In cells that are preincubated with radioactive sulfate (Na_2_^35^SO_4,_), ricin becomes labelled, indicating transport of this toxin to the Golgi apparatus. Although, dynamin is not required for ricin endocytosis [52,53], at least, it seems that transport from endosomes to the Golgi network is dynamin-dependent (Figure 3) in HeLa cells [75]. It was demonstrated that overexpression of mutant dynamin in HeLa cells did not affect ricin degradation but transport of endocytosed ricin to the Golgi and total ricin toxicity were strongly inhibited [75]. Since ricin transport after endocytosis is based on vesicles, it can be predicted that intracellular traffic of this toxin would depend on some Rab proteins, GTPases that regulate many steps of vesicular transport [76]. Moreover, knowledge about the involvement or lack of association of particular Rabs in ricin transport defines, at least partially, specific mechanisms of its intracellular routing. It seems to be the case for the Rab9-dependent pathway, that delivers proteins from late endosomes to the TGN [77,78]. Overexpression of a dominant-negative mutant of Rab9, (Rab9S21N), failed to protect cells from ricin intoxication and did not prevent sulfation of modified ricin-A-sulf-1 [79]. In addition, ricin transport to the TGN is independent of Rab-11, clathrin [79], as well as Rab7 [48]. Rab-9-independent transport of ricin supports the notion that this toxin may be transported to the Golgi complex directly from early endosomes. It has been demonstrated that isoforms of Rab6, Rab6A and Rab6A′ are important in early endosomes-TGN transport, with a special role of Rab6A′ in this pathway [80] (Figure 3). Both isoforms are expressed at similar levels in all cells. They differ in three amino acids located near one of their GTP-binding domains [81]. It was demonstrated that the ricin-related Shiga toxin B-chain utilizes direct transport from early endosomes being dependent on Rab6A′ [82]. The hypothesis that ricin may use a similar, early endosomes-TGN pathway was confirmed by experiments demonstrating that its transport to the TNG is regulated by Rab6A and Rab6A′ [83]. Moreover, ricin transport between endosomes and the Golgi network is regulated by changes in the cholesterol level. Ricin can be endocytosed by cells depleted of cholesterol [54], but transport to the TGN was strongly inhibited when the cholesterol was reduced [84]. Interestingly, ricin delivery to the Golgi complex is also calcium-dependent [85] (Figure 3). Calcium regulates many steps in intracellular trafficking, including intra Golgi transport [86]. It was observed that in cells treated with thapsigargin, which specifically inhibits the ER Ca^2+^-ATPase, ricin transport to the Golgi was increased [85]. Moreover, genome-wide RNAi screens revealed human endosome-related genes such as the ESCRT component VPS2 (CHMP2A), Rab11FIP, and Rab5c as important for ricin toxicity [84]. Ricin transport also depends on the GARP complex, the SNARE Syntaxin16 (STX16), and the Golgi-related complexes TRAPP and COG [84] (Figure 3). However, VPS35, a central component of the Retromer that is required for the formation of transport carriers at the endosomes, is dispensable for ricin endosome-TGN transport. Mentioned already above, Rab6A and Rab6A′ are human homologues of yeast Ypt6p [87]. Fluorescence-based reporter assay demonstrated that Ypt6p and its regulator Rgp1p are involved in RTA transport in yeast. Moreover, some GARP complex components (Vps51p and Vps54p) and Sft2p that enables fusion of endosome-derived vesicles with the Golgi are required for ricin traffic into cells [59]. Interestingly, Tlg2p (STX16 is a mammalian homologue of this protein) is not important for endosome-to-Golgi transport of RTA. In addition to the results described above, another simple reporter method based on the detection of changes in fluorescence emissions has been described and recently demonstrated to be useful for the identification of host cell proteins involved in intracellular RTA transport [88]. Endosome-to-Golgi transport of ricin is also regulated by cAMP signal transduction in the cell. It has been demonstrated that transport of ricin from endosomes to the Golgi network and further to the ER is controlled by the Golgi-associated regulatory subunit of protein kinase A (PKA) type II alpha isozyme in lymphocytes [89] (Figure 3). Moreover, transport of ricin to the Golgi is facilitated in human cells by hVps34 [90] (Figure 3), the only identified kinase that phosphorylates phosphatidylinositol (PI) in position 3 to produce PI(3)P. PI(3)P is needed for the vesicular localization of sorting nexins SNX2 and SNX4. All these events are required for ricin transport from endosomes to the Golgi complex [90]. 

Ricin transport to the Golgi complex can be observed by electron or immunofluorescence microscopy, whereas delivery of this toxin to the ER has never been directly visualized [91]. However, it was possible to use a genetically changed RTA containing two modifications: C-terminal Golgi-specific site for tyrosine sulfation (described above: RTA-sulf1) and three partly overlapping N-glycosylation sites (ricin-A-sulf-2) that flag an ER asparagine modification with carbohydrates [69]. It appeared that ricin-A-sulf-2 became sulfated in the TGN but also core glycosylated, indicating retrograde transport to the ER. Ricin itself does not contain an ER-targeting signal or KDEL retention sequence that would allow its interaction with KDEL receptors of the target cell and mediate toxin transport from the Golgi to the ER via coatomer protein I (COPI)-coated vesicles. However, RTB binds to resident luminal ER protein, calreticulin, a KDEL-tagged protein which indirectly allows for toxin transport to the ER [92]. Thus, calreticulin can operate as a retrograde transporter for ricin movement from the Golgi to the ER (Figure 3). However, calreticulin-deficient cells remained sensitive to this toxin, indicating that transport based on calreticulin-RTB interactions does not seem to be the main pathway for ricin traffic to the ER [92]. It was suggested that since ricin can bind glycolipids, some fraction of ricin may utilize lipid-sorting signals [93]. The second Golgi-to-ER transport pathway that was considered to be used by ricin is COPI-independent but Rab6-dependent [94]. However, it was demonstrated that the expression of the GDP-restricted mutant of Rab6A (Rab6A-T27N) did not alter ricin toxicity, suggesting that ricin is transported to the ER by a pathway that does not involve Rab6A [93]. Moreover, ricin was still toxic to cells when Rab6A and COPI were simultaneously inhibited. Thus, it was concluded that ricin can circumvent the Golgi apparatus. This hypothesis was confirmed in experiments in which the COPI protein complex was depleted of its subunit, epsilon-COP [94]. Cells were transfected with a modified ε-COP bearing a temperature-sensitive mutation. At the nonpermissive temperature, this protein become degraded, and the Golgi apparatus is changed morphologically. In such conditions, ricin could still be transported to the ER, even in the presence of brefeldin A which inhibits the binding of COPI to membranes and causes disassembly of the Golgi [95]. These results strongly suggest that ricin can bypass the Golgi stack on its way to the ER. However, it should be noted that such a pathway may be induced in the cells due to the changes enforced on these cells. Going back to the considerations about the classical Golgi-to-ER transport, it has been demonstrated by genome-wide screen that the ER-Golgi intermediate compartment protein 2 (ERGIC2) may be an important regulator of ricin transport to the ER [87]. siRNA-mediated downregulation of ERGIC2 protects cells against high doses of ricin. Consistent with these results, yeast homologue of ERGIC2 (Erv41p) and its complex partner Erv46p (mammalian ERGIC3) participates in RTA transport to the ER [59]. Both Erv46p and Erv41p are components of COPII vesicles, they form an active complex that traffics between the ER and the Golgi, being important for membrane fusion in ER/Golgi transport [96]. Additionally, the yeast reporter assay identified regulators of the ADP ribosylation factor (Arf) GTPases, Glo3p and Gea1p in ricin traffic in the cell. Arf initiates the budding of COPI-coated vesicles [97]. Two components of COPI, Sec22p and Rer1p are also important for ricin transport to the ER [96]. Rer1p is located at the Golgi membrane and operates as a retrieval receptor sending membrane proteins back to the ER [98]. Sec22p is a t-SNARE protein [99] that continuously traffics between the Golgi and the ER being involved in both anterograde and retrograde transport. Interestingly, it has been demonstrated that the mammalian homologue of Sec22p, Sec22B, is also important for ricin toxicity [87]. Thus, the components of both COPI and COPII seem to be important for ricin transport to the ER in yeast (Figure 3). Moreover, it has been suggested that COPII- and COPI-dependent ricin cycling between the ER and the Golgi is necessary for ricin dislocation to the cytosol and subsequent cytotoxicity [53]. It has been demonstrated very recently that GDP-fucose transporter residing in the Golgi, Slc35c1, and Fut9, a Golgi α1,3-fucosyltransferase, are both involved in fucosylation and are crucial for ricin toxicity [100] (Figure 3).

It should be noted that ricin A-chain can be directly delivered to the lumen of the ER by expressing a recombinant RTA version with an N-terminal signal peptide. The signal sequence is removed during RTA entry to the ER. Such an experimental approach appeared to be useful in yeasts [101,102], mammalian cells [103,104] and plants [105]. This procedure enables analyzing the events that happen after entry of the toxin into the ER, studying RTA interactions with ER-specific proteins, toxin transport from the ER to the cytosol and mechanisms of its intoxication. Directed transport to the ER is useful in yeast since yeast are deprived of galactosylated cell surface receptors [106] that are able to bind to the ricin B-chain. Thus, externally added ricin does not intoxicate yeast cells. Moreover, in mammalian cells, RTB was directed to the ER to study the formation of the disulfide bond between A- and B-chains in the ER [103]. In plants, preproricin is initially synthesized. It is composed of a single polypeptide chain of the RTA and RTB [107]. The first 35 amino acid residues of preproricin contain a 26 residue N-terminal signal sequence and a 9 residue propeptide [17]. The N-terminal signal sequence directs the transport of the nascent polypeptide across the ER membrane into the ER lumen; the 9 residue propeptide is removed after proricin transport to the vacuole [17]. Using the mature RTA (with 35 amino acid residues of preproricin) in tobacco leaf cells, it was demonstrated that a significant fraction of the newly synthesized ricin is retrotranslocated from the ER to the cytosol for degradation [105].

### 2.3. Ricin Translocation to the Cytosol

It is strongly believed that only active A-chain of ricin is translocated to the cytosol [53,69,104,108,109,110]; however, some suggestions that the whole holotoxin can be transported out of the ER have also appeared [111]. Anyway, it has been demonstrated that RTA transport to the cytosol is proceeded by reduction of the internal disulfide bond that connects the ricin A- and B-chains [103]. This reduction is catalyzed by the protein disulfide isomerase (PDI) [112], the main ER foldase that is responsible for the formation, cleavage and isomerisation of disulfide bridges [113]. It has been demonstrated that PDI interacts with the ricin B-chain and can both reduce and form the disulfide bond between ricin subunits [103]. However, it seems that the disulfide reductase activity of PDI needs to be enhanced by thioredoxin reductase (TrxR) [114] (Figure 3). In eukaryotes, two thiol-disulfide exchange systems exist: The thioredoxin system that contains thioredoxin and thioredoxin reductase [115] and the glutaredoxin system that includes glutaredoxin and glutathione reductase [116]. They catalyze fast and reversible reactions between cysteines in their active site and cysteines of their disulfide substrates using NADPH and reduced glutathione (GSH) as a source of reducing equivalents, respectively. In the case of ricin, it was demonstrated that PDI, TrxR and thioredoxin (Trx) used separately were unable to directly reduce ricin holotoxin [114]. However, PDI and Trx in the presence of TrxR and NADPH could release RTA from ricin holotoxin in vitro. PDI functioned only after pre-incubation with TrxR. The reductive activation of ricin was more efficient in the presence of glutathione [114]. Disulfide bond reduction in ricin holotoxin enables liberation of RTA, but it is also suggested that it serves to activate the catalytic activity of ricin A-chain [117]. It was demonstrated that recombinant proricin activity was dependent on its release from the mature ricin that was generated from proricin. Moreover, it was shown that another member of the PDI family, TMX, a transmembrane thioredoxin-related protein, reduces disulfide bridges in ricin holotoxin [118]. TMX can activate RTA by promoting interactions between ricin A-chain and ER proteins that facilitate transport of RTA to the cytosol prior to subsequent cell intoxication [118]. Increased ability of ricin to intoxicate cells after reductive release of RTA from holotoxin may result, at least partially, from the fact that liberated ricin A-chain can be unfolded to a certain extent in the ER. Such unfolding is probably required for RTA retrotranslocation to the cytosol through a relatively narrow ER channel. In support of this hypothesis, it was demonstrated that the introduction of an intrachain disulfide bond into the ricin A-chain significantly decreased the cytotoxicity of modified toxin [119]. This was explained by a constraint in the unfolding of RTA. Moreover, it was observed that a native A-chain is quite unstable at pH 7.0 [120]. Partially-unfolded RTA was sensitive to protease digestion and disrupted tertiary structure [120]. Thus, it was considered that ricin A-chain should be unfolded in the lumen of the ER, where it is recognized by several ER chaperones in a way similar to misfolded proteins. 

Proteins that fail to become properly folded are recognized by specific ER factors that promote their transport to the cytosol for proteasomal degradation (for review see for example Refs. [121,122,123,124]). This process is called ER-associated degradation (ERAD) [122,125]. It is believed that the ricin A-chain, similarly to other A subunits of particular toxins, utilizes ERAD in its transport from the ER to the cytosol (for review see for example Ref. [110]). However, the main difference between RTA and typical ERAD substrates is that ricin A-chain avoids effective degradation by proteasome, being instead activated in the cytosol to exert its cytotoxic effect. Still, little is known about specific ERAD factors that facilitate RTA transport out of the ER. It has been demonstrated that both the A- and B-chain of ricin interact with one of the main Hsp70 chaperone family proteins, Bip (Grp78) [126]. Despite these interactions, overproduction of BiP significantly decreased RTA transport out of the ER and protected cells against this toxin. It cannot be excluded that ricin interacts with BiP that is already engaged in a bigger protein complex that forms in the ER and inhibits ricin transport to the cytosol. It has been demonstrated very recently that BiP can form a direct complex with Grp94 (Hsp90 chaperone protein) in the absence of a substrate [127]. Moreover, this interaction is nucleotide-specific. BiP and Grp94 more efficiently interact with each other at high ADP concentrations and possess lower affinity to interaction at high ATP concentrations. It has been demonstrated that inactivation of Grp94 by a specific inhibitor protects cells against ricin [128]. Except for classical chaperones (Hsps: 40, 70, 90 and 100), the ER possesses a unique class of carbohydrate-dependent lectins that recognize different ERAD substrates both in a glycan-dependent and independent manner [122,123,129]. The most known members of these lectin chaperones are calnexin/calreticulin [122,130] and the EDEM family [131,132,133,134]. Ricin B-chain interaction with calreticulin facilitates Golgi-ER transport (see above Ref. [92]), but it is not proven that this chaperone is directly involved in ricin transport to the cytosol. This is opposite to the role of EDEM1, EDEM2 and EDEM3. It has been demonstrated that RTA interacts with all EDEMs [135,136,137,138] (Figure 3). However, the mechanisms of their action during ricin translocation out of the ER are not the same. EDEM1 probably has a higher affinity for typical misfolded proteins than for ricin [135]. Thus, it can promote its transport to the cytosol only when ER translocons are not intensively occupied by ERAD substrates. EDEM2 directly facilities ricin transport to the cytosol, which induces higher cell sensitivity to this toxin [136]; whereas overproduction of EDEM3 is not relevant for RTA translocation to the cytosol [138]. Interestingly, ricin A-chain interaction with EDEM1 and EDEM2 and consequently its translocation to the cytosol and overall cytotoxicity is related to the appropriate structure and degree of hydrophobicity of RTA [57,58]. RTA P250A (with substitution of proline to alanine at amino acid position 250) of the highly hydrophobic C-terminal region (Val245 to Val256) has a changed secondary structure to a more helical one without alternations in RTA hydrophobicity [57] (Figure 2A). On the other hand, the substitutions V245S, L248N, I252N, A253S, S246V, and A253V in this region produce RTA with decreased (RTA DHF) (Figure 2B) and increased hydrophobicity (RTA IHF) (Figure 2C), respectively [58]. Both RTA P250A and RTA DHF show significant reduction in their ability for interactions with EDEM1 and EDEM2 [57,58]. Additionally, transport of RTA P250A to the cytosol and toxicity of this modified ricin were not dependent on EDEM1 and EDEM2 overproduction. These results demonstrate that for interactions between EDEM1, EDEM2 and RTA, appropriate structure and hydrophobicity of the substrate are important. RTA with a decreased amount of *β*-sheet structures that directly resulted from increased *α*-helicality [57] and RTA with very low hydrophobicity of the C-terminal region [58] exhibit reduced interactions with EDEM chaperone proteins. Interestingly, it was demonstrated that a conformational change of RTA is crucial for its binding to the surface of the ER membrane. At the physiologically relevant temperature of 37°C, RTA loses some of its helical content and rearranges the conformational structure in such a way that it exposes its C-terminal region to the membrane interior [139]. Such an insertion into the ER membrane might be necessary for RTA translocation to the cytosol. It can be concluded from these observations that an additional limiting step in RTA P250A retrotranslocation to the cytosol (apart from lack of EDEM1 and EDEM2 assistance) might result from its inability to be subjected to additional conformational changes allowing it to be stably inserted into the ER membrane. As already mentioned, EDEM proteins can recognize glycan residues present on their substrates but can also bind other structures, e.g., hydrophobic regions or unfolded motifs. This second option seems to be important for EDEMs–ricin interactions since recombinant ricin expressed in *E. coli* that was used in the experiments, lacks oligosaccharides that are normally added to ricin A-chain derived from plants [140]. On the other hand, it was demonstrated in *S. cerevisiae* that RTA glycosylation (that occurs on asparagines 10 and 236) promotes its transport from the ER to the cytosol and increases ricin cytotoxicity as block in RTA glycosylation impairs depurination of specific adenine in 28S rRNA [141]. Moreover, in the case of ricin, a glycan signal can stabilize this toxin [142]. This is opposite to typical misfolded proteins where specific oligosaccharide recognition becomes a signal for their degradation. Ricin stabilization was demonstrated by using GFP-tagged RTA containing a point mutation (E177Q) which attenuates its cytotoxicity (GFP-RTA E177Q). This toxin, engineered with a murine signal sequence for direct co-translational delivery into the ER of the host cell, was destabilized by inactivating genes required to generate and recognize the N-glycan residues [142].

In its transport from the ER to the cytosol, ricin A-chain definitely utilizes one specific type or different classes of the ER membrane translocation channels (Figure 3). Three main types of ER translocons have been identified so far: the Sec61 complex [143,144,145,146,147], the Derlin proteins [148,149,150,151,152,153] and several ER membrane multi-spanning ubiquitin ligases, including HRD1 (Hrd1p in yeast) [154,155,156,157,158,159]. The main function of the ubiquitin ligases is connected with polyubiquitination of polypeptides emerging in the cytosol prior to their transfer for the proteasomal degradation (for review see for example Ref. [160]). However, the ubiquitin ligases can also form a translocation channel being directly involved in ERAD substrates’ transport to the cytosol [156,157]. The role of Sec61 and Derlin proteins in RTA transport out of the ER is not clear and unambiguous. It is still discussed whether these channels can be used by ricin A-chain as real translocons. It has been demonstrated that RTA can interact with Sec61α, the main component of the Sec61 complex in mammalian cells. This was shown by co-immunoprecipitation studies [108,135] and additionally demonstrated with isolated yeast ER-derived microsomes [101]. Moreover, the rate of RTA degradation was significantly decreased in yeast mutants defective in protein export via the Sec61p translocon [101]. On the other hand, genome-wide RNAi screens did not identify Sec61 as important in ricin toxicity [87]. In these experiments, Sec61 was effectively downregulated, as a mix of the single most potent siRNA against each gene of the Sec61 complex was used. In addition, gene silencing of *Sec61α* did not influence RTA transport from the ER to the cytosol in HEK293 (human embryonic kidney) cells [161]. However, it cannot be excluded that unchanged ricin A-chain translocation to the cytosol upon Sec61α downregulation might result from the existence of undefined compensatory mechanisms that direct ricin to other ER channels. In the case of Derlins, the majority of collected data indicate that these proteins are not crucial for RTA transport to the cytosol. The rate of RTA degradation was not decreased in yeast cells devoid of Der1p (mammalian Derlin-1) [101]. Cells stably transfected with dominant negative constructs of Derlin-1 and Derlin-2 treated with extrinsically added ricin [135], as well as dominant negative Derlin-1 transfected mammalian cells expressing an ER-localized RTA construct [104], did not exhibit altered ricin A-chain transport to the cytosol when compared to the control cells. Moreover, overproduction of Derlin-1 or Derlin-2 did not influence RTA dislocation to the cytosol [162]. In contrast, single Derlin protein downregulation as well as gene silencing of all three Derlins (*Derlin-1*, *Derlin-2* and *Derlin-3*) showed significant rescue against ricin intoxication [87]. The assumption that Derlins can play a specific role in ricin transport across the ER membrane was strengthened by an observation showing that two factors, UFD1L and NPLOC4, that bind to Derlins, are required for ricin intoxication [87]. However, considering the effect of Derlin-1 on overall ricin cytotoxicity, it should be noted that the ER-cytosol step is not the only one that contributes to this process, since it was demonstrated that Derlin-1 is necessary for an efficient retrograde transport of ricin from endosomes to the Golgi apparatus [163] (Figure 3). The role of HRD1 ubiquitin ligase and its cofactor SEL1L seems to be the most obvious in RTA translocation to the cytosol (Figure 3). It was shown that SEL1L is required for ricin A-chain transport to the cytosol and *SEL1L* knockdown protects cells from ricin [104]. Similarly in yeast, Hrd1 and its cofactor Hrd3p facilitate RTA transport out of the ER [164]. However, recently published results demonstrated that Hrd1p and Derl2 (mammalian Derlin-2) contribute to, but do not exert, an absolute requirement for ricin intoxication [165].

## 3. Cytotoxic Action of Ricin on Cells

### 3.1. Activation of Ricin A-Chain in the Cytosol Regulation of RTA Folding versus Degradation

**Figure 4:**
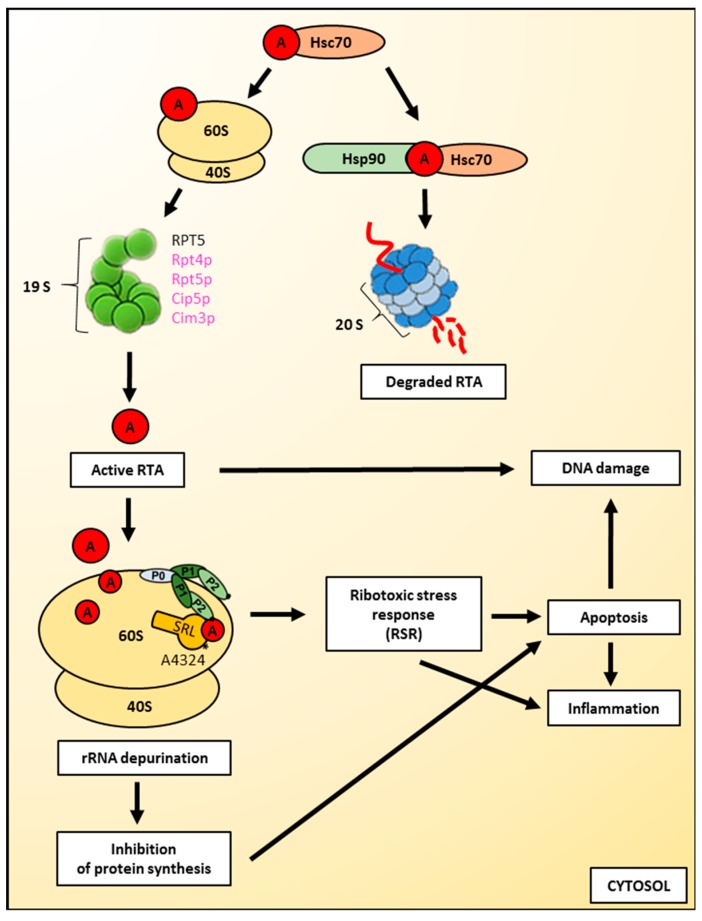
Cytotoxic action of ricin on cells. Binding to cytosolic chaperones, ribosome-mediated refolding and interactions with ribosomal and proteasomal factors are pathways by which ricin A-chain can obtain its active form. However, ricin A-chain is partially degraded by proteasomes. Active ricin A-chain is an *N*-glycosidase that removes a universally-conserved adenine at position 4324 from the α–sarcin-ricin loop (SRL) of the rRNA present in the large ribosomal subunit. The interaction of ricin A-chain with the large ribosomal subunit is facilitated by the ribosomal stalk structure, composed of P0, P1 and P2 proteins. The damage of the 28S rRNA by ricin leads to the inhibition of protein synthesis and triggers the ribotoxic stress response (RSR). Both pathways can induce apoptosis and further inflammation. Ricin A-chain can also directly induce DNA damage. Yeast proteins are shown in magenta; mammalian proteins are shown in black. 20S refers to the core particle, whereas 19S refers to the regulatory particle of the proteasome. For a detailed description, please see the text.

After translocation to the cytosol, ricin must refold into its biologically active conformation to modify its cytosolic targets (Figure 4). It is considered that there are three general pathways by which ricin A-chain can obtain its catalytic, folded structure: binding to cytosolic chaperones [53,128,166], ribosome-mediated refolding [120], and interactions with ribosomal and proteasomal factors [166]. However, it should be noted that not all of the RTA molecules translocated to the cytosol can act as an active toxin. Ricin A-chain is partially degraded by the 26S proteasome and this degradation can be blocked by specific proteasome inhibitors [57,108,135,136,167]. It seems that in mammalian cells, the majority of RTA escape proteasomal degradation [108,135]. However, in yeasts, it was shown by pulse-chase experiments that only 20% of translocated toxin appeared to be completely stable, whereas the rest was degraded during the first hour of the chase [101]. Cell fractionation has shown that this stable RTA was present in the cytosol. Interestingly, yeast proteasomes discriminate between native and structurally defective forms of RTA [164]; native RTA can avoid proteasomal degradation. This phenomenon is explained by the fact that ricin A-chain contains only two lysine residues which generally do not become efficiently ubiquitinated during toxin transport to the cytosol [109,167,168]. The lack of RTA lysine ubiqitination is common for both yeast and mammalian cells despite the fact that requirements for the ubiquitin-dependent system, Cdc48p/p97 that extracts ricin A-chain out of the ER membrane differ between these two groups. In yeast, RTA transport to the cytosol is independent of Cdc48 [164], whereas in mammalian cells, the expression of a dominant negative mutant of p97 blocked ricin toxicity and increased the time required for RTA transport to the cytosol [169]. The second general mechanism that allows toxins to avoid proteasomal degradation might be connected with their ability to obtain its fully-folded structure relatively quickly after transport to the cytosol. It has been demonstrated that folded proteins do not become proteasomal substrates even if they possess many lysine residues on their surface [170]. However, in the case of ricin, it was shown that it cannot refold spontaneously after thermal denaturation in vivo [120]. At 37°C, ricin has a conformation similar to a molten globule, and it was impossible to obtain its native state by manipulation of the buffer conditions or by the addition of a stem-loop dodecaribonucleotide or deproteinized *E. coli* rRNA, both of which are substrates for ricin A-chain. Thus, RTA is considered to be a toxin with a slow refolding rate. This rate is much slower than for example the A subunit of the cholera toxin (CTA1) that displays lower detectable sensitivity to degradation by the proteasome when compared with ricin [171]. 

The interactions of ricin A-chain with ribosomal proteins and cytosolic chaperones are crucial in gaining its proper conformation. These interactions also facilitate RTA transport to the cytosol. It has been demonstrated that an ATPase subunit of the 19S proteasome cap in yeast, Rpt4p [164], and two other proteins of the cap, Cim3p and Cip5p [101], are important for ricin A-chain transport from the ER. However, no obvious requirement for this transport was observed for the other Rpt subunits, Ubr1p or the proteasome core itself [164]. It should be noted that Rpt4p can cooperate with Cdc48p in the extraction of an endogenous substrate from the yeast ER [172]. Chaperone-like activity of ribosomes were demonstrated in experiments showing that partially-unfolded ricin A-chain was able to obtain full catalytic activity in the presence of salt-washed ribosomes [120]. The ATPase subunit RPT5 of the 19S proteasome cap prevents aggregation of denatured RTA and enhances the recovery of catalytic activity of ricin A-chain in vitro [166]. In addition, it was shown in vivo that Rpt5p is required for maximum toxicity of RTA dislocated from the yeast ER. Interestingly, the anti-aggregation properties of the 26S proteasome are independent of its proteolytic activities [166]. Cytosolic chaperones protect and activate ricin A-chain but they also have a much broader spectrum of action, as they regulate the general fate of RTA dislocated from the ER [128]. The mechanisms of cytosolic chaperones activity include ricin A-chain binding to Hsc70 (cytosolic member of Hsp70 family). It was shown that Hsc70 prevents aggregation of the heat-inactivated toxin and can recover its catalytic activity. Inhibition of cytosolic Hsc70 protected HeLa cells from ricin [128]. However, the concentration of Hsc70 co-chaperones may regulate the amount of RTA that can gain the catalytic activity and the fraction that will be degraded. Interaction of the RTA-Hsc70 complex with BAG-2 and Hip induces RTA activity in vitro, promoting the sensitivity of cells to ricin. On the other hand, co-chaperones BAG1 and CHIP facilitate ricin A-chain destabilisation. CHIP is an E3 ubiquitin ligase that interacts not only with Hsp70 but also with another type of cytosolic chaperone, Hsp90. Moreover, another dual co-chaperone, Hop, is an Hsp70-Hsp90 organizing protein. It reversibly links Hsp70 and Hsp90 by recruiting Hsp90 to the existing Hsp70-substate protein complex. This promotes transfer of the substrate from Hsc70 (Hsp70) to Hsp90 [173]. Overexpression of Hop decreases sensitivity to ricin, suggesting that sequential interaction of RTA with Hsc70 and Hsp90 directs the toxin to inactivation and destabilisation [128]. Consistent with this hypothesis, inhibition of Hsp90 sensitized cells to ricin [128]. RTA inactivation may be mediated by an ubiquitination process. This mechanism is not fully elucidated, but it was suggested that a low amount of RTA can be ubiquitinated in the cytosol. In yeast, ubiquitination occurs via an unknown E3 ligase [164]. It has been demonstrated that RTA is not ubiquitinated by Hrd1p during dislocation [164], but introduction of additional lysyl content into RTA reduces its cytotoxicity by increasing ubiquitin-mediated proteasomal degradation [168]. In yeast, no significant changes in the growth rate were observed in cells lacking individual Hsp40, Hsp70 and Hsp90 family members or the Hsp70 and Hsp90 co-chaperones [164].

### 3.2. Ricin A-Chain Action on Ribosomes

Ricin A-chain is an *N*-glycosidase that removes a universally-conserved adenine at position 4324 in mammalian cells (Figure 4) and A3027 in yeast from the α–sarcin-ricin loop (SRL) of the rRNA present in the large ribosomal subunit ([5,8,10,11,174] and see Introduction for details). This disables the binding of specific elongation factors to the ribosome and inhibits protein synthesis [9,16,17]. It is known that ricin A-chain influences the structure of the ribosomal RNA. It alters the dynamic flexibility of the GTPase activating centre of the ribosome, which part is SRL. These conformational changes disturb the transition between the pre-and post-translocational states of the elongation cycle [175]. As earlier mentioned in this article, ricin does not depurinate *E. coli* ribosomes. However, its inability to exert a cytotoxic effect on prokaryotic ribosomes does not result from the fact that RTA is incapable to act on the 23S rRNA from the large bacterial ribosomal subunit. In fact, ricin A-chain depurinates the sarcin-ricin loop of naked 23S rRNA [176], suggesting that for the proper catalytic activity of ricin, the whole ribosome and particularly ribosomal proteins are crucial [177]. It has been demonstrated that the ribosomal stalk structure facilitates the interaction of ricin A-chain with the large ribosomal subunit in eukaryotes [177,178,179] (Figure 4). The human ribosomal stalk structure is composed of three types of phosphoproteins, P0, P1 and P2. They are assembled into a pentameric protein complex comprised of a single P0 protein bound by two heterodimers of P1 and P2 proteins [180,181]. It has been shown recently that P1–P2 proteins represent a primary binding site for RTA [182], with a more critical role for the P1B–P2A dimer [183]. Interestingly, the stalk structure differs significantly between prokaryotic and eukaryotic ribosomes. In prokaryotic cells, the stalk proteins exhibit very little sequence homology to eukaryotic counterparts. Thus, it was suggested that they are solely functionally analogous to eukaryotic proteins [184]. This important observation significantly contributes to our understanding of species specificity for ricin and ultimately defines the sensibility of the SRL for depurination by this toxin. The ribosomal stalk is a dynamic structure located in proximity to the SRL. It was suggested that ricin A-chain binding to the stalk proteins may allow RTA to be placed directly by the sarcin-ricin domain, which facilitates more efficient detection of the rRNA substrate by RTA [177]. Based on the analysis of interactions between RTA and the stalk proteins, a two-step binding model was proposed with the first step characterized by a slow association and dissociation rate, and a second step with much faster rates of interactions [185,186]. Within this model, the binding of RTA to the stalk was then more specifically divided into four phases. In the first phase of binding, ricin A-chain is concentrated on the surface of the ribosome and directed to the stalk [185,186]. This step is based on slow and nonspecific electrostatic interactions. In the next phase, RTA interacts with the stalk through more specific and stronger electrostatic interactions, which are saturable. Step three represents the delivery of RTA to the SRL [185,186]. This transfer is mediated by the C-terminal domain (CTD) of the stalk proteins. Their involvement in this process decreases the possibility of RTA dissociation out of the ribosome and leads to rapid recruitment of the toxin by SRL. Finally, in the fourth phase, ricin A-chain specifically depurinates the α-sarcin-ricin loop [185,186]. Recently published results demonstrated that the C-terminal domain of P1 is the main docking site for RTA as deletion of P1 CDT but not P2 CDT decreased the affinity of the stalk structure for ricin A-chain [182]. However, studies of another group have demonstrated that RTA mainly recognizes the highly conserved C-terminal tail of P2 (residues 106–115) in which two residues, Leu and Phe, are critical for the interaction with RTA [187]. These residues might also be important for RTA binding to other P proteins. Moreover, the crystal structure of RTA with P2 protein shows that GFGLFD motif of the C-terminal P2 is inserted into a hydrophobic pocket of RTA, suggesting that the flexibility of the P2 peptide interaction with ricin A-chain is based on hydrophobic rather than electrostatic interactions [188]. It was shown before that seven arginine residues located at the RTA/RTB interface are involved in ricin A-chain interaction with the ribosome [189,190]. In the holotoxin structure, each arginine residue is covered by RTB, completely or only partially [191]. Nevertheless, this causes the ribosome binding site on RTA to be blocked by RTB, thereby disabling ricin holotoxin to depurinate ribosomal rRNA. Thus, holotoxin RTA can become catalytically active only after its release from RTB which results in revealing the ribosome binding site [192,193]. Interestingly, RTA with a modified ribosome binding site is less toxic than a variant with lower catalytic activity but unchanged ribosome binding activity [194]. It has been demonstrated that by introducing R189A/R234A and R193A/R235A double mutations, ricin A-chain binding to the stalk stimulates ribosome depurination by orienting the active site of RTA toward the SRL, thereby allowing docking of the target adenine into the active site [192]. Recently published results also showed that Arg235 of ricin A-chain serves as a main interacting residue with ribosomes and cooperates with nearby arginines to allow RTA to interact with the stalk with fast kinetics in order to achieve the binding specificity necessary for SRL depurination [191]. It was initially proposed, based on a study with analytical ultracentrifugation, that RTA interacts with the 60S ribosomal subunit with a molar stoichiometry of 1:1 [195]. However, a model involving conformational changes is currently preferred [196]. In this model, after binding of RTA to the ribosome, conformational rearrangements of both RTA and ribosomes occur, allowing the formation of a high affinity complex. These conformational changes directly influence the catalytic activity of ricin A-chain. It has recently been shown that the flexibility of the α-helix (residues 99–106) of RTA is connected with the regulation of the depurination activity by ricin A-chain, which directly influences the rate of protein synthesis inhibition [197]. Moreover, it was proposed that the flexibility of the α-helix could affect the side chain orientation of Glu-177, which is critical for the depurination activity of ricin [198,199]. Interestingly, ricin A-chain is not able to bind to the isolated 40S ribosomal subunit, however, its rRNA and/or ribosomal proteins may promote optimal and stable interactions of ricin with the whole ribosome, since binding of RTA to 80S ribosomes was approximately 3.5-fold stronger than binding to the isolated 60S subunits [177,195]. The interaction of ricin A-chain with the ribosomal stalk structure has been evaluated as a potential drug target [200]. Several peptides (3 to 11 amino acids in length) corresponding to the C-terminal end of P proteins were examined in their ability to interact with RTA and block its catalytic activity. It appeared that a four amino acid peptide is the shortest one that can inhibit depurination activity of RTA by preventing toxin binding to the ribosome [200]. 

It has been demonstrated that one molecule of ricin A-chain is able to inactivate over 1500 ribosomes per minute in a cell-free preparation of ribosomes [18,19]. The multiplexed digital droplet (ddPCR) assay showed that depurination events in lung cell cultures can be detected as early as 1 h after ricin treatment (1 nM) and within 9 h of exposure the maximum ribosomal damage of 70% was reached [201]. This effect was sustained for at least 24 h post-exposure. However, it should be noted that depurination in cell-based systems would be expected to occur at a lower rate than in cell-free systems. In the cell, the whole A-B holotoxin is applied and there is a lag time that is required for toxin uptake and its intracellular transport until the rRNA substrate is reached. Depurination rates are difficult to compare between in vivo and in vitro experiments, moreover, the rates of depurination may differ between cell types [176,201,202]. Nevertheless, it can be assumed that ricin-induced depurination is a very rapid enzymatic process.

### 3.3. Mechanisms of Ricin-Induced Apoptosis

Despite the very effective ricin-mediated rRNA depurination event that results in the inhibition of protein synthesis, it cannot be stated that these processes by themselves lead to cell death [24,30,31,32,33,203]. It has been demonstrated that ricin can induce apoptosis, autophagy and release of cytokine inflammatory mediators [25,26,27,28,29,30,204] (Figure 4). These processes have been studied for over two decades with a growing number of results that indicate their significance for cell death observed. However, it is still unclear whether the inhibition of protein synthesis is sufficient to induce apoptosis in all cell lines and to what extent other factors are required for the induction of apoptosis [30,205]. 

Early reports describing that ricin is capable of inducing cell death by apoptosis came from in-vitro studies that correlated cellular morphological changes with apoptosis [26,206,207,208] and from in-vivo studies in which epithelial, endothelial and myeloid cells were used [204,209,210,211]. It was observed that cells treated with ricin exhibit chromatin condensation, membrane blebbing, rounding of the cells, formation of apoptotic-like bodies, and DNA fragmentation that are considered to be typical markers of apoptosis [26,27,206,207,209,210]. Moreover, it has been reported that intracellular targets of ricin are not limited only to the ribosomal RNA. Deproteinized (naked) RNA, synthetic oligoribonucleotides, nuclear and mitochondrial DNA, polyA, tRNA and viral nucleic acids were published to be depurinated by purified ricin and other type II RIPs [211]. These ricin activities were often classified as actions not directly connected with apoptosis. It has been reported that the early DNA damage observed in human endothelial cells HUVEC in parallel to the arrest of protein synthesis was not a consequence of ribosome inactivation or apoptosis but results from direct action of ricin on DNA [212] (Figure 4). Li and Pestka [213] suggested that ricin and other RIPs may induce rRNA damage, in addition to the classical way, also through increased expression and activation of host RNases. Moreover, ricin-mediated rRNA depurination might facilitate toxin interaction with one or both dsRNA-binding domains of a kinase associated with the ribosome, PKR (double-stranded RNA-activated protein kinase), thereby causing the activation of PKR [214]. PKR plays a role in interleukine-8 (IL-8) induction, which may trigger the ribotoxic stress response (RSR) (see below). 

It is considered that the main mechanisms of ricin-dependent apoptosis are based on the activation of caspases [215,216,217,218,219,220,221,222], Bcl-2 family members [223,224,225], and stress-associated signaling pathways [102,213,215,219,226,227,228,229,230,231,232,233,234,235,236,237,238]. The mechanisms of ricin-induced cell death promoting pathways are also connected with direct and indirect action of ricin on DNA [212,239], ricin-mediated reactive oxygen species production [24,215,218,224,240,241,242], and ricin B-chain-induced apoptosis [33,243].

#### 3.3.1. Ricin-Induced Activation of Caspases

It is assumed that apoptosis can be activated via two major pathways, extrinsic and intrinsic. The extrinsic or receptor-mediated mechanism includes ligation of the death receptors that stimulate the activation of the initiator caspase-8, which then triggers downstream events by direct stimulation of caspase-3 or cleavage of the protein Bid. In the intrinsic pathway, mitochondria function as the main operation center. Damage to mitochondria results in outer membrane depolarization and permeabilization (MOMP) which triggers the release of several proapoptotic factors, including cytochrome c. This leads to the activation of caspase-9, which then triggers effector caspase-3 [244,245]. Poly (ADP-ribose) polymerase (PARP) is one of the best described substrates for caspase-3 which can cleave 116kD PARP into 85 and 31kD fragments [246]. PARP cleavage is one of the most studied hallmarks of apoptosis. It is believed that mitochondria and the intrinsic pathway of apoptosis activation are critical in signaling for cell death in ricin-intoxicated cells. It has been reported that in ricin-treated cells, a loss in mitochondrial membrane potential, rapid release of cytochrome c, activation of caspase-9 and caspase-3, and DNA fragmentation were observed [215,216,217,218,219,220,221,222]. Caspases may be profoundly involved in the pathway, resulting in DNA fragmentation [220,247]. Moreover, ricin-dependent PARP cleavage has been observed in different cell lines including HeLa [218] and U937 cells [216,220]. Interestingly, studies performed in U937 cells might, at least partially, answer the question about correlation between different ricin-mediated death mechanisms in cells. In ricin-treated U937 cells, intracellular NAD(+) and ATP levels were decreased and this reduction was followed by ricin-mediated protein synthesis inhibition [220]. The PARP inhibitor, 3-aminobenzamide (3-ABA), blocked the depletion in NAD(+) and ATP levels. Significant PARP cleavage was observed more than 12 h after ricin addition, while DNA fragmentation reached a maximum level within 6 h of incubation [220]. Thus, it was concluded that the PARP cleavage is not an early apoptotic event associated with the induction of ricin-mediated apoptosis and that the pathway leading to cell lysis via PARP activation and NAD(+) depletion is independent of the pathway leading to DNA fragmentation. Moreover, it seems that human BAT3 (HLA-B-associated transcript 3, Scynthe) [248] is an important regulator of ricin-dependent caspase-3-mediated apoptosis [215,221]. It interacts with ricin A-chain, which was confirmed by co-immunoprecipitation and confocal microscopy studies. BAT3 possesses at its C-terminal end, a canonical caspase-3 cleavage site, thus being the substrate for this protease. As a result of this cleavage, a 131 amino acid C-terminal fragment of BAT3 (CTF-131) is generated. It was observed that ricin-mediated induction of cell apoptosis by caspase-3 activation resulted in BAT3 cleavage after 4 h treatment with ricin. On the other hand, ricin-induced apoptosis was significantly reduced in cells with a decreased level of BAT3. Importantly, CTF-131 but not BAT3 was responsible for the observed direct ricin-induced apoptotic morphological changes such as: cell rounding, nuclear condensation, and phosphatidylserine exposure [221]. It was also observed that internalized ricin co-localized with endogenous BAT3 in the nuclei of HeLa cells. It was suggested that caspase-3-mediated proteolysis of BAT3 may require caspase-3 translocation to the nucleus. Another important regulatory factor is the mitochondrial intramembrane protein AIF (apoptosis-inducing factor). BAT3 interacts with AIF in the cytosol [248], regulates its stability by inhibiting proteosomal degradation and also induces nuclear translocation of this factor. AIF may regulate caspase activation, however, after translocation to the nucleus, it is involved in chromatin condensation and DNA fragmentation [248]. 

As described above, the majority of experiments promote the idea that ricin initiates the intrinsic, mitochondrial-dependent pathway of apoptosis. However, some findings suggest that the extrinsic pathway might be also important in ricin-induced apoptosis. It has been demonstrated by TUNEL immunohistochemical staining, flow cytometry, and Western blotting that purified ricin A-chain was able to induce apoptosis in mouse embryonic fibroblast (NIH 3T3), by activation of caspase-8 and -3 but not caspase-9 [222].

#### 3.3.2. Activation of Bcl-2 Family Members by Ricin

Bcl-2 (B-cell lymphoma protein-2) family members regulate the intrinsic pathway of apoptosis. This family of proteins consists of three subfamilies playing opposing functions in this process: proapoptotic BH3-only members (Bim, Bid, Puma, Noxa, Hrk, Bmf, and Bad), proapoptotic effector molecules (Bax and Bak), and antiapoptotic Bcl-2 family proteins (Bcl-2, Bcl-xL, Mcl1, A1, and Bcl-B) [244,245,249]. It has been shown that overexpression of Bcl-2 improves the growth of MCF-7 breast cancer cells treated with ricin by 10-fold [223]. However, ricin retained its ability to inhibit protein synthesis in those cells. It has also been demonstrated that the ricin-induced apoptosis of hepatoma cells, BEL7404, results from increased expression of Bak and decreased levels of Bcl-xl and Bax [224]. However, overexpression of Bcl-2 can protect BEL7404 against ricin. These results are in agreement with other observations, supporting the view that signaling through mitochondria can represent the main mechanism of ricin-induced apoptosis. It is possible that in cells with an elevated level of Bcl-2, ricin-induced cell death is inhibited through titrating the function of its pro-apoptotic homologues, such as Bax. Consistent with these results, in cells overexpressing Bcl-2, a lower level of ricin-induced caspase-3 activity and PARP cleavage were observed in comparison to control BEL7404 cells treated with ricin [224]. Interestingly, the use of a caspase-1-specific inhibitor also partially blocked ricin-induced apoptosis, implicating a role for caspase-1, and therefore possible involvement of the inflammasome and cytokine production in this process. Other studies showed that BER-40 cells (brefeldin A-resistant mutant cell line of Vero) were highly resistant to ricin-induced apoptosis as compared with their none-modified counterparts, parental Vero cells [225]. It was suggested that the function of mitochondria may be somehow altered in BER-40 since a lack of release of cytochrome c was observed in these cells. However, the number and structure of mitochondria were not changed in these cells. Also, the expression level of Bcl-2 (which is the regulatory protein involved in the release of cytochrome c), was the same in Vero and BER-40 cells treated with ricin. Thus, it was suggested that relatively early apoptotic signaling pathways prior to those that lead to the release of cytochrome c may be altered in BER-40 cells. However, there is a possibility that mitochondria-related factors such as Bax and Bcl-xl, or the regulation of Bcl-2 activity by phosphorylation and dephosphorylation, might also be involved in the apoptosis resistance phenotype in BER-40 cells [225].

#### 3.3.3. Activation of Stress Associated Signaling Pathways by Ricin

It has been proposed that the damage of the 28S rRNA by ricin triggers a specific kinase-activated pathway termed the ribotoxic stress response (RSR) [226]. In this signaling pathway, stress-activated protein kinase SAPK/JNK1 (c-Jun N-terminal kinase) is activated together with its activator kinase SEK1/MKK4 [250]. It has been demonstrated that this activation does not result from the inhibition of protein synthesis, but is directly connected with signaling from the 28S rRNA affected by ricin. JNKs, p38 and extracellular-receptor kinases (ERKs) belong to the Ser/Thr kinases termed MAPK (mitogen-activated protein kinase) family [251]. It is known that ricin can activate not only JNK, but also ERK and p38 MAPK in RAW 264.7 macrophages, and this is necessary for further activation of a variety of proinflammatory mediators [227]. Interestingly, not only ricin holotoxin but also high concentrations of ricin A-chain were able to induce both the p38 and JNK MAP kinase signaling pathways; however, signaling through the JNK kinase appeared to be more important in inducing the apoptotic response by RTA in the nontransformed epithelial cell line, MAC-T cells [219]. Ricin treatment induces the expression of proinflammatory cytokines and chemokines such as TNF-α, interleukin (IL)-1, IL-6, and IL-8 [227,228,229,230,231]. It was demonstrated that macrophages and IL-1 signaling play a central role in the inflammatory process triggered by ricin [232]. Moreover, ricin is an activator of the NALP3 inflammasome, a scaffolding complex that mediates pro-IL-1β cleavage to active IL-1β by caspase-1 [233], (for review see also Ref. [234]). The proinflammatory response of ricin is believed to be initiated by phosphorylation of the kinase ZAK, a MAP3K, that is located upstream to the kinases p38 MAPK and JNK in a signal transduction pathway leading to proinflammatory gene expression [235]. It has been demonstrated that the JNK and p38 pathways regulate the expression of cytokines and downstream transcription factors in a different way [227]. The use of specific chemical inhibitors of the SAPK pathways in ricin-treated RAW 264.7 macrophages showed that suppression of the p38 pathway almost completely inhibited IL-1α and -β expression, while blocking the JNK pathway increased the expression of these cytokines. In contrast, inhibition of both pathways equally attenuated the ability of ricin to induce TNF-α gene expression [227]. Moreover, the role of p38 in ricin-induced expression of various proinflammatory genes was demonstrated [229], and chemical inhibition of the p38 pathway in the human monocyte/macrophage cell line 28SC blocked ricin-induced IL-8 secretion [228]. In addition, inhibition of the p38 MAPK pathway in ricin-treated RAW 264.7 macrophages attenuated both TNF-α secretion and apoptosis [236]. It was concluded that the ribotoxic stress response may trigger multiple signal transduction pathways through the activation of p38 MAP kinase. These results underline the major role of MAPK kinases and especially p38 in ricin-dependent regulation of apoptosis and in proinflammatory signals gene expression. 

Besides MAPKs activation, ricin can also trigger the NF-κB pathway that is responsible for regulation of the expression of genes encoding inflammatory and pro-coagulant mediators [215,230]. It was suggested that the inhibition of protein synthesis by ricin may lead to the activation of NF-κB. Moreover, the activation of both the JNK and p38 MAPK pathway as well as NF-κB occurs independently. Inhibition of TNF-α in cultured primary human airway epithelial cells did not prevent ricin-induced activation of NF-κB [230]. However, inhibition of NF-κB resulted in the release of cytochrome c from the mitochondria [252] and JNK1 kinase activation [252,253], suggesting an anti-apoptotic function of this transcription factor. The regulation of survival and apoptotic signals triggered by MAPKs and NF-κB in response to ricin remains to be determined. 

The second stress-associated signaling pathway affected by ricin is the unfolded protein response (UPR), which is related to the ER stress. This signaling pathway can be characterized as a cell reaction to the accumulation of unfolded or misfolded proteins in the lumen of the ER [122,124,159,254]. The UPR is regulated by three ER transmembrane receptors: the RNA-dependent protein kinase like ER kinase (PERK); the inositol-requiring ER to nucleus signal kinase-1 (IRE1) and the activating transcription factor-6 (ATF6) (for review see e.g., Refs. [124,255]). It has been demonstrated that ricin inhibits activation of the UPR in yeast by preventing *Hac1* mRNA splicing [102]. The *Hac1* mRNA is an important regulator of the IRE1 signaling pathway. Activated yeast Ire1p triggers unconventional splicing of the *Hac1* mRNA leading to the synthesis of a transcription factor that specifically binds to promoters containing unfolded protein response elements [256]. Moreover, it was shown that RTA-mutated forms that could depurinate ribosomes but did not cause yeast cell death were unable to inhibit activation of the UPR by the ER stress-inducer tunicamycin [102]. These results suggest that the inability to activate the UPR in response to the ER stress contributes to the cytotoxicity of ricin. Other investigations also showed that ricin A-chain enhanced its own cytotoxicity by inhibiting the UPR [237]. In human epithelial cell lines (HeLa and MAC-T), RTA inhibited both phosphorylation of IRE1 and splicing of *XBP1* mRNA (homologue of yeast *Hac1*) induced by tunicamycin. However, in contrast to these studies, it was demonstrated that ricin can activate PERK and ATF6 of the UPR pathways, but not the IRE1 branch [238]. This led to cell growth arrest and apoptosis. It was proposed that blocking of the UPR response allows RTA to trigger cell death through a mechanism that is independent of protein synthesis inhibition.

#### 3.3.4. Direct Action of Ricin on DNA and Ricin-Mediated Inhibition of DNA Repair Enzymes

As already mentioned in this review, not only rRNA but also DNA is a ricin substrate in the catalytic reaction mediated by this toxin [212]. It has been demonstrated that ricin and other RIPs can act on DNA and many different polynucleotidic substrates, releasing adenine from the sugar phosphate backbone of poly- and polydeoxynucleotides [257]. It was even suggested that RIPs should be classified as polynucleotide:adenosine glycosidases. The nuclear DNA injury revealed in cultured cells by the alkaline-halo assay and the alkaline filter elution technique was attributed to adenine release from RNA-free chromatin [212] and naked DNA [257]. Interestingly, in the case of ricin, the DNA damage was observed very early after cells treatment with ricin, and this damage was concomitant with the protein synthesis inhibition. At this time, the annexin V binding assay, caspase-3 activity, changes in cell morphology, and the formation of typical apoptotic DNA fragments were not detectable. It was suggested that ricin damages DNA in a way that does not result from ribosome inactivation or apoptosis [212] (Figure 4).

Ricin can also act indirectly on DNA by the inhibition of a DNA repair pathway. It has been demonstrated that this toxin, as well as other RIPs, releases an adenine from the ADP-ribosyl group of PARP [239]. NAD^+^-dependent auto ADP-ribosylation of PARP is necessary for its active involvement in the DNA repair pathway called base excision repair [258]. It was suggested that depurination of auto-modified PARP by ricin results not only in the inhibition of DNA repair, but also leads to further ADP-ribosylation of PARP and depletion of the intracellular levels of NAD^+^ and ATP. This, together with the impaired repair of damaged DNA, would cause cell necrosis induced by lethal amounts of ricin [239]. Moreover, it has been reported that ricin can inhibit the repair of H_2_O_2_ and the alkylating agent methyl methane sulphonate (MMS)-induced DNA lesions in HUVEC and U937 cells [259]. The inhibition of DNA repair by ricin seems to result from direct interactions with the DNA repair machinery. Importantly, ricin concentration used in the experiments to inhibit DNA repair was not sufficient to cause direct DNA damage or to induce total protein synthesis inhibition.

#### 3.3.5. Ricin-Mediated Reactive Oxygen Species Production

The production of the reactive oxygen species (ROS) in cells has been reported to be involved in apoptosis induction by activating signal transduction mechanisms located upstream of the caspase-3 signalization pathway [260]. These pathways may be regulated by the changes in the oxidation status of the proteins involved in apoptosis signaling. It has been demonstrated that ricin increases the ROS levels in both yeast [24] and human cells [218]. The studies carried out on yeast suggested that the production of ROS is a necessary and sufficient condition for ricin-mediated induction of apoptosis [24]. Free radicals are scavenged by reduced glutathione (GSH). However, it was demonstrated in ricin-treated HeLa [218] and U937 cells [240] that the level of GSH was decreased. Thus, it was suggested that the GSH loss takes place downstream of caspase activation during the ricin-induced apoptotic process [240].

Interestingly, it has been demonstrated that the ROS formation is dependent on the presence of both extracellular and intracellular Ca^2+^ [261]. A rapid elevation of cellular calcium levels was observed in ricin-treated hepatoma cells [224]. Madin-Darby Canine Kidney (MDCK) cell death was significantly blocked by 1,9-deoxyforskoIin (DDF) treatment, a drug that can reduce ion flux through several ion channels. This protective effect was significantly reversed by the increase in the extracellular Ca^2+^ concentrations [241].

Ricin-induced apoptosis is correlated not only with the elevation of the level of calcium ions. It was demonstrated that in ricin-treated U937 cells, the level of intracellular Zn^2+^ was increased and zinc was much more redistributed into the cytosol [242]. This occurs as an early apoptotic event, and exogenously-added Zn^2+^ inhibited the ricin-induced apoptosis. It was suggested that Zn^2+^ ions play a regulatory role in ricin-mediated apoptosis through their dissociation/association with certain intracellular elements.

#### 3.3.6. Ricin B-Chain-Induced Apoptosis

Studies carried on U937 cells have demonstrated that the interaction of ricin B-chain with membrane glycoproteins and glycolipids may trigger signaling events leading to apoptosis [33]. This lectin activity-dependent mechanism was distinct from apoptosis signaling pathways induced by ricin A-chain. It has been demonstrated that carboxymethylated-(CM-) ricin B-chain was responsible for DNA fragmentation and typical apoptotic nuclear morphological changes, which were very similar to those observed in ricin-treated cells [33]. CM-ricin B-chain failed to inhibit protein synthesis in U937 cells. Thus, these experiments support the hypothesis that ricin-induced apoptosis or at least some of the apoptotic pathways are independent and not correlated with protein synthesis inhibition, at least in U937 cells. Recently published results have also shown that ricin-induced apoptosis is not solely attributed to the A-chain [243]. The intact heterodimeric ricin and ricin chains were injected into rats in order to study ricin-induced apoptosis in liver, which is a major site of in vivo ricin uptake and cytotoxicity [262]. It has been demonstrated that ricin was responsible for the intrinsic apoptosis pathway since increased cytochrome c content, activation of caspase-9 and caspase-3, and enrichment of DNA fragments in the cytosol were observed [243]. These authors observed also the B-chain in the cytosol and reported that it caused cytochrome c release from mitochondria in vivo and in vitro. These results suggest that a direct interaction of ricin B-chain with the mitochondrial outer membrane can be involved in ricin-induced apoptosis. The involvement of recombinant RTB in macrophage activation has also been studied [263]. It was demonstrated that RTB stimulated inducible nitric oxide (NO) synthase (iNOS) and TNF-α and IL-6 expression, which are involved in the activation of protein tyrosine kinase, NF-κB and JAK-STAT signaling.

## 4. Perspectives

### 4.1. Ricin-Based Immunotoxins

In the 19th century, Paul Ehrlich proposed the “magic bullet concept”, which states that drugs can directly enter target cells and hit only abnormal cells of the human body [264]. Since then, the idea of a selective action of drugs that are able to affect only specific types of cells has been dynamically developed. However, the specificity of this process is challenging. The concept is utilized particularly in the field of cancer therapy with the application of immunotoxins (ITs) [265,266,267,268]. ITs are chimeric proteins composed of a toxin or a part of the toxin conjugated with a monoclonal antibody (mAb) or its fragment. When toxins are coupled to other carriers: growth factors, hormones or lectins that preferentially bind to some cell types, they are more commonly referred to as “chimeric toxins” or “conjugates” [269]. Ricin is the most commonly used plant toxin in the construction of ITs [30,269]. The first ricin-based ITs were prepared by binding holotoxin to a specific mAb [270]. Despite high efficiency, a large non-specific toxic effect of these immunotoxins was observed and made them impossible to use in a clinical setting. In a different experimental approach, only the A-chain of ricin was used, but also such ITs exhibited non-specific toxicity [271], due to the fact that receptors present on many cell types can recognize mannose residues present on the RTA [272]. To solve this problem, new ITs were prepared with deglycosylated RTA (dgRTA) [269,273,274]. 

Ricin-based immunotoxins are promising in the treatment of many types of diseases. Autoimmunity, immunodeficiency and neoplasia are examples of diseases connected with deregulation of the immune system. These dysfunctions are characterized by changes in the normal amount or function of Th (helper) cells. ITs composed of RTA and cell-reactive antibodies can specifically target neoplastic cells. It was shown that treatment of Th cells with Fab’ fragments of anti-L3T4 antibody bound with RTA (Fab’anti-L3T4-A) inhibit keyhole limpet hemocyanin (KLH)-specific Th cells from proliferation and differentiation of the antigen-specific B cells (trinitrophenyl-(TNP)-specific B cells) [275]. These results indicate that Fab’anti-L3T4-A is able to specially inhibit Th cells that activate B cells. Another immunotoxin RTA-4D5-KDEL was constructed by connecting the anti-HER2 single chain variable fragment 4D5 scFv and KDEL, the ER-targeting peptides, with the C-terminal part of the RTA. Experiments showed that RTA-4D5-KDEL had a strong inhibitory effect on the ovarian cancer cells, SKOV-3, which were HER-2 overexpressing, and caused little damage to H460 lung cancer cells and to kidney HEK 293 cells. The KDEL of the RTA-4D5-KDEL immunotoxin was able to direct the recombinant protein to the ER. In light of this information, it can be assumed that this immunotoxin has a strong inhibitory effect on ovarian cancer cells with overexpression of HER2, and that it will exhibit little toxicity in normal cells [44]. Bladder cancer is one of the most frequent tumors. This disease is treated with transurethral resections and additionally with local immunotherapy or chemotherapy with good results; however, there is no ideal therapy to heal invasive carcinoma. A new antibody-based immunotoxin BCMab1-Ra was generated by linking of BCMab1-, a novel mouse monoclonal antibody, specific for aberrantly glycosylated Integrin a3b1 in this type of cancer with the ricin A-chain (Ra) [276]. The effect of the BCMab1-Ra on bladder cancer was investigated on a 57-year-old patient that refused radical surgery and chemotherapy. It has been demonstrated that the use of BCMab1-Ra first reduced the tumor, and that after 30 weeks of treatment there was no tumor observed by cystoscope examination. Moreover, human anti-mouse antibody (HAMA) that would indicate a strong immunologic response was not detectable in the blood circulation of this patient [276].

Various therapies are being utilized in the treatment of cancer. Traditional procedures such as radiation therapy, chemotherapy and surgery have some limitations and give serious side effects. Immunotoxins represent another technique with the possibility to increase the selectivity of action, but further development in this field is required.

### 4.2. Ricin Conjugated with Nanoparticles

During recent years, nanoparticles (NPs) have been studied intensively both as carriers used for delivery of therapeutic drugs (conventional drugs, recombinant proteins, vaccines and nucleotides) to certain cells and as therapeutic agents that may act per se or modulate activity of other compounds [277,278,279,280]. One carrier that can deliver (NPs) to cells is the ricin B-chain. The internalization mechanism as well as intracellular transport of ricinB:Quantum dot (QD) nanoparticle conjugates have been studied in different cells [41,281,282]. It was concluded that Qdots may have severe consequences on cell physiology [281,282]. Moreover, the internalization of ricinB:QDs in HeLa cells is dependent on dynamin and based on a macropinocytosis-like mechanism [41]. 

A complex of carbon dots (CDs) with RTB has been evaluated for enhanced immunomodulatory activity of RTB [283]. It was demonstrated that CDs-RTB can facilitate macrophage proliferation and increase the generation of nitric oxide (NO), IL-6 and TNF-α in RAW 264.7 cells, indicating enhanced immunomodulatory activity of CDs-RTB in comparison to RTB acting alone [283]. 

Another interesting example is a recombinant version of a ricin nanoparticle (T22-mRTA-H6) containing the T22 peptide, an efficient ligand of the cell surface marker CXCR4 (a cytokine receptor selectively overexpressed in metastatic cells of many cancer types) at the amino terminus followed by a mutated version of the ricin A chain and a hexahistidine tail at the carboxy terminus [284]. In this construct, mutation N132A was introduced to suppress the vascular leak syndrome (see below); a furin cleavage site was incorporated to allow the release of the N-terminal region in the endosome as well as a KDEL motif was added to mediate retrograde transport. Interestingly, this construct was engineered in order to allow for ricin A-chain aggregation and to become a targeting agent for the precise tumor delivery of protein-only nanoparticles. The recombinant T22-mRTA-H6 was produced in *E. coli* and purified. The spontaneous formation of self-assembled nanoparticles was possibly due to the combination of the cationic peptides at the amino terminus and polyhistidines at the carboxy terminus. T22-mRTA-H6 nanoparticles show highly selective therapeutic effects, and ricin A-chain was highly active on target cells, significantly reducing the effect of leukemia cells on relevant organs.

### 4.3. Vaccines against Ricin and Neutralizing Antibodies against Ricin

Despite numerous medical applications in which ricin can be used, this toxin is among the most potent and lethal substances that are known [35,285]. Currently, no approved vaccine or therapeutics exist to protect against ricin intoxication. The idea to develop a preventive vaccine against ricin has grown over the last years mainly because of the increasing concern that crude ricin powder can easily be made and used as a bio-threat agent. Two of the leading vaccine antigen candidates, the closely related RTA-based subunit vaccines, RiVax™ and RVEc™, are now under development [39,286,287]. RiVax™ is a full-length recombinant derivative of RTA whose enzymatic activity has been largely eliminated through a point mutation in a key active site residue (Y80A). RiVax™ also contains a mutation in the site (V76M) attributed to the induction of the vascular leak syndrome (VLS) [286]. The VLS is the main side-effect of ricin-derived immunotoxins. It has a complex etiology involving damage to vascular endothelial cells [288]. Mutation in the vaccine to alter the VLS motif was introduced to eliminate this toxicity. RVEc™ is a truncated derivative of RTA that lacks the hydrophobic carboxy-terminal region (residues 199–267) as well as a small hydrophobic loop in the N-terminus (residues 34–43). RVEc™ mutations do not directly influence the active site of RTA, but the removal of both regions causes that ricin present in this vaccine is inactive with reduced ability to cause the vascular leak syndrome [39,287]. Both candidate vaccines are under investigation in animal studies and Phase I clinical trials. Furthermore, the results of two Phase I clinical trials have indicated that RiVax™ is safe and immunogenic in humans [289,290]. One obvious strategy to augment the overall immunogenicity of vaccines is the use of next-generation adjuvants. However, adjuvants themselves may not be sufficient to achieve maximal immunogenicity. Enhancing the immunogenicity of vaccines may require a structure-based redesign of the antigen itself. The resulting combinations of mutations led to the identification of derivatives of RiVax which are several times more efficient [291].

In the late 1880s, Paul Erhlich and others first described the potential use of antibodies (Abs) to completely inactivate the toxin. Immunity to ricin is associated with the production of protective antibodies. Since those early studies, many studies of antisera and antibody preparations derived from different animal species and tested on a diversity of cell types have been made. Anti-RTA and RTB antibodies were tested in rabbits and mice and displayed some neutralization action. Some results suggest that antibodies neutralize ricin by perturbing toxin uptake and/or intracellular trafficking without affecting the toxin attachment to cell surfaces. This confers passive immunity in vivo [37,291,292,293]. On the other hand, blocking ricin attachment to receptors on the cell surface is the mechanisms of action of other specific antibodies (24B11 and VHH D10/B7) [294]. Multiple studies revealed that there are three general classes of ricin-specific Abs; those that bind RTA, RTB and ricin holotoxin [286,295]. As part of an effort to engineer ricin antitoxin and immunotherapies, libraries of phage-displayed, heavy chain-only antibodies (V_H_Hs) have been produced and well characterized [296]. It has been demonstrated that immunity against ricin is mediated by antibody. However, the specificity of particular epitopes involved in protective immunity remains unclear. The importance of toxin-neutralizing antibodies in protection against ricin is not questioned. However, the exact correlation between the structure of RTA and the induction of protective immunity must be more strictly evaluated. In just the past 10 years, several reports have been published that demonstrated that passive administration of a toxin-neutralizing antibody is sufficient to display mice protection to a lethal dose of ricin delivered by injection, ingestion or inhalation [292,297,298,299,300,301].

## 5. Concluding Remarks

Ricin can be considered as a powerful tool to study intracellular pathways in general and cell death mechanisms that include apoptosis, inflammation or cell stress-induced signaling. On the other hand, exact knowledge about ricin action in the target cells is necessary to produce effectively working ricin-based immunotoxins or vaccines. One of the most interesting discoveries that has been made recently describes a vital sugar code for ricin toxicity, that is conserved from mouse to human [100]. This mechanism is based on defined glycosidic structures that determine cellular fate upon exposure to the toxin. The question of whether depurination of rRNA is necessary for ricin-induced cell death is still being discussed. It is believed that in addition to rRNA damage, ricin can induce apoptosis, inflammation and DNA damage. The correlation between these processes has been intensively studied. This knowledge is constantly being expanded as the huge contribution to this field has been made over the past years. An old dogma about ricin has currently been investigated. It was believed that a single A-chain molecule of ricin or other type-2 RIPs have the ability to kill one eukaryotic cell [20]. However, it was recently reported that one or a few molecules of ricin A-chain present in the cytosol is not sufficient to inhibit protein synthesis [302]. Moreover, cells with a partial inhibition of protein synthesis can, upon ricin removal, increase the level of protein production and survive the toxin challenge. Thus, in contrast to the previously accepted model, ongoing toxin delivery to the cytosol appears to be necessary for the death of cells exposed to sub-optimal ricin concentrations [302]. It was also suggested that ricin and other RIPs can be more toxic to cancer cells than to normal cells, due to the higher rate of protein synthesis in malignant cells during proliferation or due to the changes in receptor concentration on their surfaces or altered intracellular transport of the toxin [30,42,303,304,305]. This is, however, not always the case (for review see e.g., Refs. [30,285]). Thus, for specific delivery of ricin to cancer cells, directing ricin to particular epitopes on tumor cells is necessary. On the other hand, some specific properties of ricin may enhance its effect on cancer cells. The ability of RTA to inhibit UPR may make it more potent in targeted therapy for cancer. It has been demonstrated that an increased level of spliced *XBP1* relatively to unspliced *XBP1* correlates with poor prognosis in breast cancer [306], and XBP1 has been proposed as a therapeutic target for solid tumors [307]. Thus, ricin treatment may be particularly useful in cancer cells where UPR is already accelerated by conditions such as hypoxia. These findings highlight the role of ricin as a valuable component of modern immunotoxins.
